# In vivo and in vitro genotoxicity of *N*-nitrosopyrrolidine following UVA irradiation

**DOI:** 10.1186/s41021-025-00334-y

**Published:** 2025-05-26

**Authors:** Yusuke Hanaki, Sakae Arimoto-Kobayashi

**Affiliations:** Graduate School of Medicine, Dentistry and Pharmaceutical Sciences, Okayama University, Okayama, 700-8530 Japan

**Keywords:** N-nitrosamine, Photomutagenicity, Photogenotoxicity, Mammalian cell micronucleus test, Mammalian erythrocyte micronucleus test, Ames test

## Abstract

*N*-nitrosopyrrolidine (NPYR) is a volatile nitrosamine that is thought to be a human carcinogen. It is found in air, wastewater, food, and feed. Photo-activation of NPYR can occur as it drifts through the environment. We previously found that NPYR irradiated in phosphate buffer was directly mutagenic without metabolic activation or simultaneous irradiation. Here, we aimed to determine NPYR activity after UVA irradiation. The mutagenic activity of irradiated NPYR was relatively stable, and ~ 23% of it persisted after 168 h of storage at 37 °C. Micronuclei (MN) were also found without metabolic activation in human-derived keratinocytes (HaCaT cells) after NPYR irradiation in vitro and the peripheral blood reticulocytes (PBRs) of mice with inhibited cytochrome-P450-mediated metabolism then injected with irradiated NPYR in vivo. The active photoproduct of NPYR is thought to be genotoxic to bone marrow, resulting in MN formation in PBRs. The action spectrum of MN formation in PBRs exposed to NPYR irradiated with monochromatic light was plotted along the absorption curve. The production ratio of active NPYR photoproduct followed the NPYR absorption curve. Genotoxicity becomes systemic with externally irradiated NPYR that penetrates the skin or when NPYR is irradiated just under the skin and enters the systemic circulation. Risk analyses of public health-related volatile *N*‐nitrosamines generated via environmental photoactivation including NPYR, should be considered.

## Introduction

**Fig. 1:**
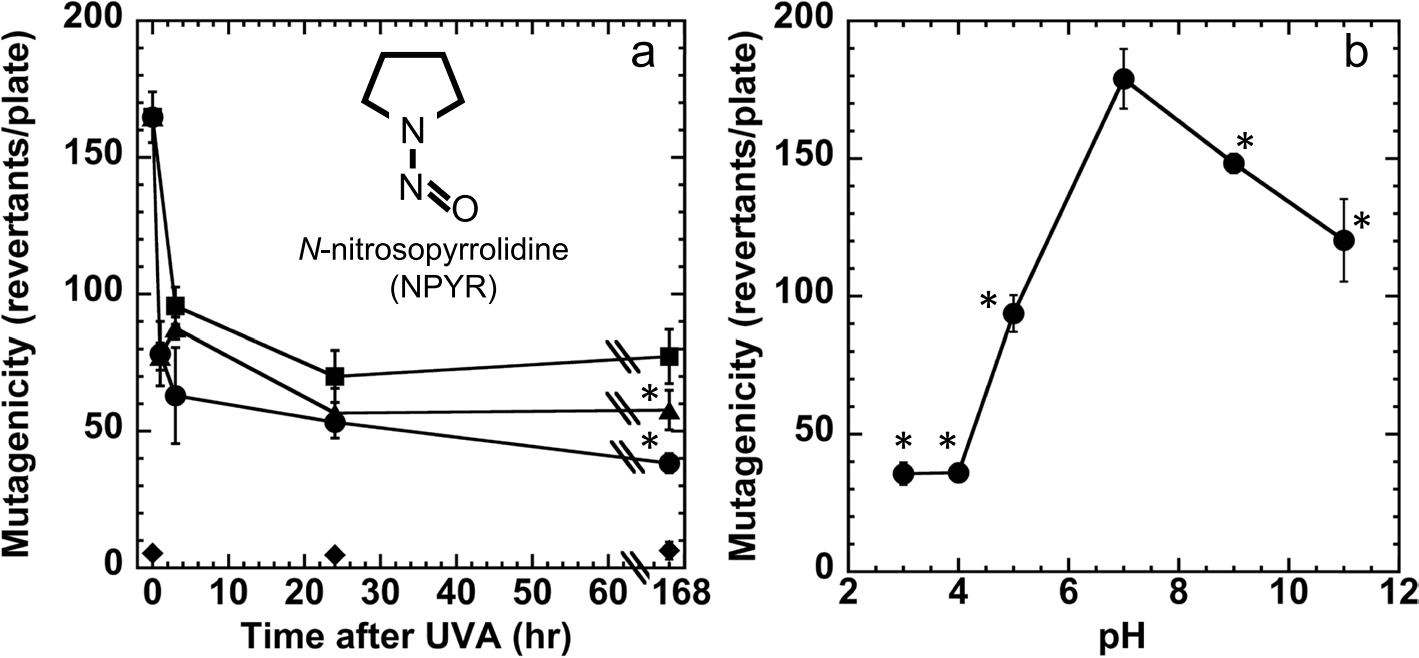
Effects of storage and pH changes on the stability of photomutagenicity. **a** Chemical structure of *N*-nitrosopyrrolidine (NPYR) and stability of the mutagenicity of UVA (20 kJ/m^2^)-irradiated NPYR (0.1 mM) after storage for 1, 3, 24 and 168 h kept at 37 °C (circles), 4 °C (triangles), or −20 °C (squares). Treatment with non-irradiated solvent (PBS) assayed at 1, 24 and 168 h is shown (diamond) as the negative control. *p < 0.05, significantly different from those at 37 °C or 4 °C. **b** Mutagenicity of UVA (20 kJ/m^2^)-irradiated NPYR (0.1 mM) following treatment at pH 3, 4, 5, 9, or 11 at 37 °C for 1 h. **a**, **b** The assay was performed using *Salmonella typhimurium* TA1535 without metabolic activation. Experiment was repeated twice, and the standard deviation (SD) is indicated by the error bar (*n* = 3). *p < 0.05, significantly different from those at pH 7

The Report on Carcinogens lists 256 substances, including nitrosamines [1], such as *N*-nitrosopyrrolidine (NPYR; CAS 930–55-2, Fig. 1a). NPYR is thought to be a human carcinogen produced in foods contaminated with nitrite, and it is a volatile nitrosamine found in the air while cooking, tobacco, waste-water, and food and feed. Exposure can occur through vapor inhalation during cooking or ingestion [2]. NPYR mainly induces liver tumors in rats, and some of its identified DNA adducts have been found in human tissues [3]. The key carcinogenic activation pathway is α-hydroxylation, and P450IIE1 is the most active P450 species in NPYR metabolism [4, 5]. We previously found that ultraviolet A (UVA) irradiation activates NPYR into mutagenic products that do not require metabolic activation [6]. NPYR is photoactivated in the presence of phosphate, formate or sulfate [7]. Another nitrosamine, *N*-nitrosomorpholine is also photo-activated in the presence of phosphate, acetate, propionate, succinate, citrate, phenyl phosphate, or ATP [7, 8]. Contaminants such as airborne phosphate might photoactivate NPYR as it drifts in the environment and lead to genotoxicity when it falls onto humans. We previously identified the structure of the direct-acting mutagen, *N*-nitroso-1-phosphonooxypyrrolidine in UVA-irradiated NPYR in the presence of phosphate [6]. Cytochrome P450 enzymes (CYPs) metabolize NPYR to active and inactive compounds [9]. The CYP-metabolism of UVA-irradiated NPYR remains unknown. Synthetic N-nitroso-1-phosphonooxypyrrolidine is inactivated by phosphatase [7]. Furthermore, NPYR and near-UV radiation induce chromosomal aberrations in Chinese hamster lung cells [10]. We hypothesize that UVA irradiation from sunlight activates NPYR suspended in the air or water containing phosphate, creating directly active genotoxic products that might remain stable for several days and remain suspended in the environment, causing direct genotoxic damage to human cells, such as skin or organ cells. We also hypothesized that NPYR irradiation just under the skin would create an irradiated form of NPYR that circulates systemically.

Here, we characterized the stability and pH tolerance of UVA-irradiated NPYR. We also investigated the in vitro genotoxicity effects of the directly active products derived from NPYR after UVA irradiation in human-derived keratinocytes (HaCaT) in vitro and on micronuclei (MN) formation in peripheral blood reticulocytes (PBRs) in mice in vivo.

## Materials and methods

### Materials

We obtained the following materials from the respective vendors: NPYR (Nacalai Tesque Inc., Kyoto, Japan), 1-aminobenzotriazole (ABT) (Cayman Chemical, Ann Arbor, MI, USA), *N*-ethyl-*N*-nitrosourea (ENU) (Sigma-Aldrich corp., St. Louis, MO, USA), *N*-methyl-*N*-nitrosourea (MNU) (FUJIFILM Wako Chemicals, Osaka, Japan), and the human keratinocyte cell line (HaCaT) (CLS Cell Line Service GmbH, DKFZ, Heidelberg, Germany). *Salmonella enterica* subspecies I*,* serovar *Typhimurium* (*Salmonella typhimurium*) strain TA1535 [*hisG46 ΔuvrB gal bio chl1005 rfa1001*] was a kind gift from Dr. B. N. Ames at the University of California, Berkeley [11]. All other reagents were obtained from commercial sources. The absorption spectra of NPYR were assessed using a U-2001 spectrophotometer (Hitachi High-Tech Co., Tokyo, Japan).

### Animals

Mice (Slc: ICR male, 5-week-old, average weight 25–27 g) were purchased from Japan SLC Inc. (Hamamatsu, Japan). Five mice were housed per cage in the animal room and randomly separated into treatment groups at least 1 week before the commencement of experiments. The mice had free access to murine chow pellets (MF; Oriental Yeast Co. Ltd., Tokyo, Japan) and water, and were maintained on a 12-h light/12-h dark cycle with optimum air exchange and a constant room temperature of 20 °C. All experiments were performed in accordance with the Guidelines for Animal Experiments of the Okayama University Advanced Science Research Center (permission nos. 2012214, 2,015,046, 2,018,032, and 2,021,556) based on the Act on Welfare and Management of Animals (Act of Japan, No. 105 of October 1, 1973, and the Amendment of Act No. 68 of 2005) and Standards Relating to the Care, Management, and Alleviation of Pain and Distress of Laboratory Animals (Notice of the Ministry of the Environment No. 88, 2006).

### Preparation of UVA-irradiated NPYR and detection of photomutagenicity using the Ames test

Reaction mixtures containing 0–0.1 mM NPYR in 20 mM sodium phosphate buffer (pH 7.4) were placed in ice-cold trays (Nunc, Roskilde, Denmark) and exposed to UVA light (20 or 50 kJ/m^2^, unless otherwise stated). We used soft glass and plastic plate covers to exclude UV-B radiation (< 320 nm). The light intensity determined using a UVX Radiometer (UVP Inc. Company Seven; Montpelier, MD, USA) was 1,020 ± 170 (µW/cm^2^) at 365 nm. The UV dose (kJ/m^2^) used in each experiment was regulated by adjusting the length of exposure. Thereafter, the irradiated NPYR samples were stored at −20 °C until use. The dose of irradiated NPYR was calculated based on the original NPYR concentration before irradiation.

We examined the mutagenic stability of NPYR irradiated with UVA. NPYR solution (0.1 mM) dissolved in phosphate-buffered saline (PBS) was irradiated with UVA (20 kJ/m^2^). The irradiated samples were placed in microtubes wrapped with black paper and aluminum foil to protect them from light and stored at 37 °C, 4 °C, or −20 °C for 0, 1, 3, 24, and 168 h. The samples were assayed using the Ames test without metabolic activation [11]. The positive and negative controls were MNU (2 µmole) and PBS, respectively. All experiments were repeated twice.

We then assessed the effects of the irradiated NPYR on pH 3, 4, 5, 7, 9, or 11adjusted with HCL (0.1 M) or NaOH (0.1 M), and allowed to stand for 1 h at 20 °C. The pH of the samples was adjusted to 7 using HCL (0.1 M) or NaOH (0.1 M), and the samples were assayed using the Ames test with *S. typhimurium* TA1535, without metabolic activation.

### Detection of in vitro MN formation in HaCaT cells treated with irradiated NPYR

An in vitro micronucleus assay was performed as previously described [12], following the advice provided by Dr. Akihiro Wakata, Drug Safety Research Laboratories, Astellas Pharma Inc. Japan, based on a guidebook [13] and the Organization for Economic Co-operation and Development (OECD) guidelines [14]. Briefly, HaCaT cells (9 × 10^4^ cells/3 mL) were seeded in wells containing Dulbecco’s modified Eagle’s medium (DMEM) supplemented with 10% fetal bovine serum (FBS) and incubated at 37 °C with 5% CO_2_ for 24 h. The cells were washed with PBS, then gently shaken at 37 °C in the dark with 3 mL of irradiated NPYR (0–80 M previously irradiated with 20–50 kJ/m^2^ UVA) for 1 h. The irradiated NPYR was removed, then the cells were washed with PBS and incubated in fresh medium for 44–46 h. Thereafter, the cells were harvested using trypsin–EDTA. Cell numbers were counted microscopically using a hemocytometer and cell survival was determined. The cell suspension was centrifuged at 200 × g for 5 min at 20 °C. The resulting cell pellet was then resuspended and fixed in a mixture of methanol and acetic acid (3:1). A drop of the cell suspension was placed on a clean glass slide and air dried. The cells were stained by mounting with 40 g/mL acridine orange solution and immediately observed under a fluorescence microscope (Olympus BX60, Olympus, Tokyo, Japan) equipped with a B filter. A total of 1,000 cells were observed. The number of cells with MN was counted, and the ratio of the number of cells with MN to a total number of cells (hereafter referred to as cells with MN [%]) was calculated. As a positive control, cells were treated with 8-methoxypsolaren (4 ng/mL) and UVA (50 kJ/m^2^). All experiments proceeded in triplicate, and the standard deviations (SDs) are indicated by error bars (*n* = 3).

For the dose–response experiments, NPYR was dissolved in PBS at 0–80 M and irradiated or not with UVA at 50 kJ/m^2^. Cells were treated with irradiated or non-irradiated NPYR for 15 min, and MN formation was evaluated. To determine the treatment-time-dependence of MN formation, NPYR was dissolved in PBS at 20 M and irradiated with UVA at 50 kJ/m^2^. Cells were treated with irradiated NPYR for 5, 10, 15, 30, and 60 min and then washed, after which MN formation was evaluated. Negative control cells were treated with PBS for 60 min.

### In vivo MN formation in the peripheral blood reticulocytes of mice treated with irradiated NPYR

The in vivo micronucleus assay was performed as previously described [15, 16], along with valuable advice provided by Dr. Akihiro Wakata, based on OECD guidelines [17]. Mice (ICR, male, 6 weeks old, average weight 28–30 g) were divided into three groups of five mice each. NPYR was dissolved in PBS (0.1 mM) and irradiated or not with UVA at 20 kJ/m^2^.

In Experiment 1, Group A received a single intraperitoneal (IP) injection of non-irradiated NPYR (0.5 mL), Group B received a single IP injection of UVA-irradiated NPYR (0.5 mL), and Group C received a single IP injection of 0.5 mL of ENU (25 mg/kg body weight [BW]), as a positive control. At 0, 24, and 48 h after the injection, 5 µL of peripheral blood was collected from the tail arterial vein. No anticoagulants were administered. Blood samples were placed on acridine-orange-coated glass slides and immediately covered with coverslips. All experiments were performed in triplicates. The sample slides were examined by fluorescence microscopy within a few days. The number of peripheral blood reticulocytes (PBRs) with greenish-yellow fluorescent MN per mouse (hereafter referred to as the number of PBRs with MN) was recorded. One thousand reticulocytes were measured from each animal.

In Experiment 2, ABT dissolved in saline was injected intraperitoneally into mice in all three groups 1–3 (100 mg/kg BW) 2 h before irradiated-NPYR injection [18]. Mice in Group 1 received a single IP injection of 0.5 mL of non-irradiated NPYR, mice in group 2 received a single IP injection of irradiated NPYR (0.5 mL), and mice in Group 3 received a single IP injection of 25 mg/kg BW ENU dissolved in saline (0.5 mL), as a positive control. The number of PBRs with MN was recorded. One thousand reticulocytes were measured from each animal.

### Monochromatic irradiation

Monochromatic irradiation was performed using the Okazaki Large Spectrograph at the National Institute for Basic Biology (Okazaki, Aichi Prefecture, Japan). The UV dose (20 kJ/m^2^) at each wavelength was equalized by adjusting the irradiation time. Mixtures (4 mL) containing NPYR (0.1 mM, final) in sodium phosphate buffer (20 mM, pH 7.4) were exposed to monochromatic radiation at 300, 320, 340, 360, 380, or 400 nm at 25 ºC. Following irradiation, the samples were stored at −20 °C until use. The formation of MNs in PBRs of mice treated with monochromatically irradiated NPYR was analyzed as described above.

### Statistical analyses

Data are expressed as the means ± SD for each data point, as indicated in each figure. The SDs are represented as error bars. Statistical analyses were performed using KaleidaGraph (Synergy Software, Reading, PA, USA) and Excel statistics (SSRI Co. Ltd., Tokyo, Japan). Statistical significance was set at *p* < 0.05.

## Results

### Effects of storage and pH changes on the stability of photomutagenicity of irradiated NPYR

The effects of storage time on the mutagenicity of the irradiated NPYR are shown in Fig. 1a. During the first hour, the mutagenicity of the irradiated NPYR decreased to almost 50%. From 1 to 168 h, the mutagenicity of the irradiated NPYR decreased slightly. However, after 168 h (7 days) of storage, approximately 23% (kept at 37 ºC), 35% (kept at 4 ºC), and 47% (kept at −20 ºC) of the direct-acting mutagenicity of the irradiated NPYR was retained (Fig. 1a). The stability of the mutagenicity of irradiated NPYR was slightly dependent on the storage temperature, and the direct-acting mutagenicity was significantly higher for samples stored at −20 ºC than at 4 ºC or 37 ºC, and for those stored at 4 ºC than at 37 ºC (Tukey's test). Hereafter, irradiated NPYR was stored at −20 ºC.

Treatment at pH 3–5, 9, or 11 for 1 h significantly decreased the mutagenicity of irradiated NPYR compared with pH 7 (Dunnett's tests; Fig. 1b). When treated at basic pH (9 and 11), the mutagenicity of irradiated NPYR decreased to approximately 83% and 67%, respectively. When treated at acidic pH values of 3, 4, and 5, the mutagenicity of irradiated NPYR decreased to approximately 20%, 20%, and 52%, respectively (Fig. 1b).

### MN formation in HaCaT cells treated with irradiated NPYR

**Fig. 2:**
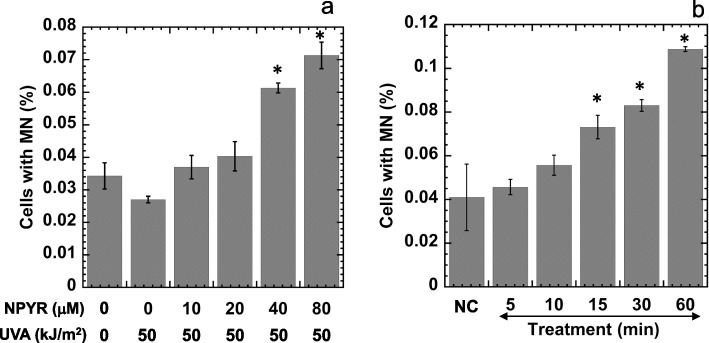
Genotoxicity of irradiated NPYR toward HaCaT cells. **a** Dose–response of MN formation after exposing HaCaT cells to irradiated NPYR. NPYR (0–80 M) was dissolved in PBS and irradiated with UVA at 0 or 50 kJ/m^2^. Cells were treated with irradiated NPYR for 15 min, and MN formation was evaluated. *p < 0.05, significantly increase compared with NPYR (0 M) plus UVA (0 kJ/m^2^). **b** Treatment-time dependence of the formation of MN in cells exposed to 20 M NPYR irradiated with 50 kJ/m^2^ UVA. *p < 0.05, significantly increased compared with negative control (NC). **a**, **b** Experiments were repeated twice, and the SD is indicated by the error bar (*n* = 3)

The cells with MN (%) dose-dependently increased in cells treated with irradiated NPYR, and the cells with MN (%) significantly increased in cells treated with 40 M and 80 M irradiated NPYR than those treated with irradiated solvent (50 kJ/m^2^) or non-irradiated, as determined using Dunnett's test (Fig. 2a). The cells with MN (%) treated with 8-methoxypsolaren and UVA (50 kJ/m^2^) was 0.117 ± 0.024 (positive control). The cells with MN (%) increased in a time-dependent manner (Fig. 2b). The cells with MN (%) treated with irradiated NPYR (NPYR 20 M with 50 kJ/m^2^ UVA) for 15, 30, and 60 min was significantly higher than the cells with MN (%) in the NC group.

### In vivo MN formation in PBRs of mice treated with irradiated NPYR

**Fig. 3:**
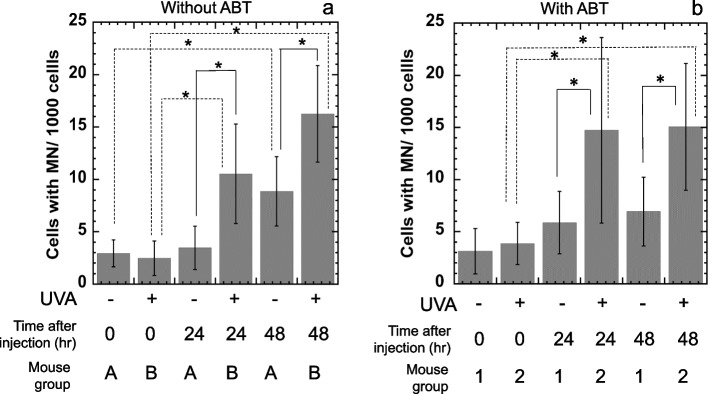
MN formation in the PBRs of mice treated with UVA-irradiated or non-irradiated NPYR. NPYR (0.1 mM) was irradiated with UVA (0 or 20 kJ/m^2^), and the irradiated solution (0.5 mL) was intraperitoneally injected into mice. Samples of peripheral blood were collected after 0, 24 and 48 h, and MN formation was evaluated. **a** Experiments were performed without 1-aminobenzotriazole (ABT) pretreatment. **b** Two hours prior to the experiments, ABT (100 mg/kg body weight) was intraperitoneally injected into mice. (ab) The experiment was repeated twice, and the SD is indicated by the error bar (*n* = 5). *p < 0.05, significantly different from each other

The in vivo genotoxicity of irradiated NPYR was determined based on MN formation in PBRs of mice intraperitoneally (i.p.) injected with irradiated NPYR, as in Experiment 1 (Fig. 3a). At 0 h after injection, the number of PBRs with MN in Groups A and B did not differ significantly. At 24 h after injection, the number of PBRs with MN per mouse in Group A did not increase compared to that at 0 h, but the number in Group B significantly increased compared to that at 0 h. At the 48 h after injection, the number of PBRs with MN was significantly higher in Group B than in Group A, and the number in Group B significantly increased from 0 to 24 h. However, at the 48 h after injection, the number of PBRs with MN in Group A was also significantly increased compared to that at 0 h. The number of PBRs with MN in the positive control group (Group C) at 0, 24, and 48 h after injection was 0.83 ± 0.85, 31.1 ± 8.13, and 17.7 ± 5.39, respectively.

It is likely that some of the original NPYR remained in the irradiated NPYR samples and were activated by cytochrome P450 s in mice to form MN in PBRs, with or without UVA. Hence, ABT was injected into mice prior to the irradiation with NPYR to suppress cytochrome P450 s, and MN formation in PBRs of mice injected with irradiated NPYR was examined as in Experiment 2 (Fig. 3b). After ABT pretreatment, mice with non-irradiated NPYR treatment (Group 1) showed no significant difference in the number of PBRs with MN at 0, 24, and 48 h after injection. In contrast, the number of PBRs with MN in the treated group with irradiated NPYR treatment (Group 2) increased significantly and in a time-dependent manner from 0 to 24 h and 48 h. The number of PNRs with MN at 24 h and 48 h after injection was significantly higher in Group 2 than in Group 1. The number of PBRs with MN in the positive control group (Group 3) at 0, 24, and 48 h after injection was 2.53 ± 1.73, 32.1 ± 8.65, 30.4 ± 8.88, respectively.

### Action spectra of in vivo MN formation in peripheral reticulocytes

**Fig. 4:**
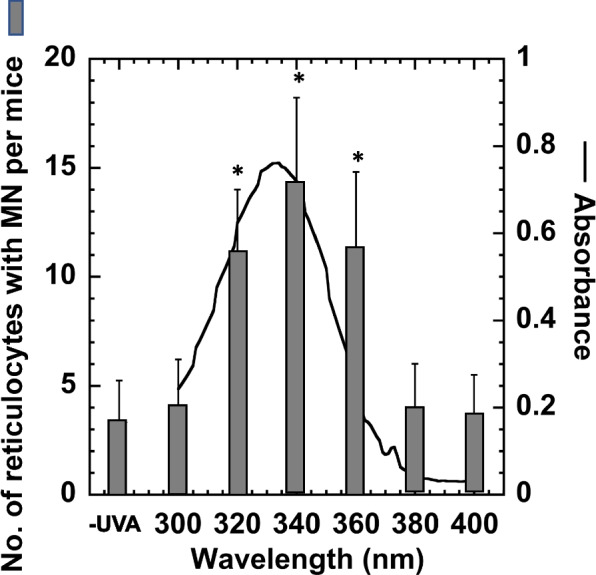
Action spectra of MN formation following treatment with NPYR (0.1 mM) irradiated with different wavelengths of monochromatic light (20 kJ/m^2^ each). Two hours after ABT injection, irradiated NPYR (0.5 mL of each) was injected into mice. After 0, 24, and 48 h, the number of peripheral reticulocytes with MN in 1,000 reticulocyte was counted, and the data at 48 h are shown (square). The UV absorption spectra of NPYR (5 mM) was measured in 20 mM sodium phosphate buffer at pH 7.4 (line). The experiment was repeated twice, and the SD is indicated by the error bar (*n* = 15). *p < 0.05, significantly different from the negative control (without UVA)

The action spectra of MN formation in PBRs of mice treated with irradiated NPYR were examined (Fig. 4). NPYR was irradiated with monochromatic light at 300, 320, 340, 360, 380, or 400 nm, and the irradiated NPYR was injected into mice. The number of PBRs with MN did not increase 0 h after injection with irradiated NPYR. However, at 24 h and 48 h after injection, the number of PBRs with MN significantly increased in mice treated with NPYR irradiated with 320, 340, or 360 nm monochromatic light compared with those treated with non-irradiated NPYR (Fig. 4). The number of PBRs with MN was highest after treatment with NPYR irradiated with light at 340 nm, which is the absorption maximum of NPYR. Treatment with NPYR irradiated with shorter or longer wavelengths (300, 380, or 400 nm) did not significantly increase the number of PBRs with MN. At 380 and 400 nm, NPYR showed no UV absorbance, and the number of PBRs with MN did not increase (Fig. 4). Thus, the spectrum of action for the effects on the number of PBRs with MN in mice treated with irradiated NPYR followed the NPYR absorption curve (Fig. 4).

## Discussion

The bioactivation of *N*-nitrosodimethylamine (NDMA) catalyzed by P450 2E1 leads to the formation of α-hydroxy-NDMA, and the mutagenic intermediate is unstable and spontaneously decomposes to generate reactive nucleophiles or to break it down [19]. During NPYR metabolism, produced α-hydroxylation of NPYR occurs via microsomal CYP450, followed by subsequent cleavage to generate unstable intermediates [20]. However, *N*-nitroso-1-phosphonooxypyrrolidine formed after NPYR was irradiated with UVA in phosphate-containing solution [6]. We found that the activity of irradiated NPYR was relatively stable, as ~ 23% of its activity remaining after 168 h of storage at 37 ºC (Fig. 1a). We isolated *N*-nitroso-1-phosphonooxypiperidine produced by irradiating *N*-nitrosopiperidine with UVA and found that its activity was similarly stable during 30 min of incubation at 37 °C at pH 7.0 [21]. Under phosphatase reactions or acidic conditions, the de-esterification of *N*-nitroso-1-phosphonooxypiperidine may generate short-lived active products that react with DNA [21]. The activity of irradiated NPYR was affected by the pH, and the mutagenicity of irradiated NPYR was stable under neutral conditions (Fig. 1b). Hence, irradiated NPYR might maintain relatively stable activity when suspended in environmental air. However, the active components may react with DNA when they reaches cells or tissues, resulting in genotoxic effects. The intrusion of volatile nitrosamines through the skin surface is a possible route of entry into the body. *N*-nitrosomorpholine (NMOR) and *N*-nitrosodiethanolamine penetrate rat skin to maxima of ~ 34% and 78%, respectively [22], and irradiated NMOR induced MN formation in HaCaT cells [16].

The present study found that irradiated NPYR was genotoxic in HaCaT cells and that MN formation was dose-dependent (Fig. 2). Volatile NPYR might adhere to environmental particulate matter and irradiated with environmental light including UVA in phosphate-containing solution. Photo-activated NPYR attached to these suspended particles might contact with the human skin, resulting in genotoxicity. Another possibility is that adhered NPYR adsorbs onto the skin and become irradiated with sunlight under the skin surface to produce an active compound of irradiated NPYR, which may enter the circulation.

MN formation significantly increased in PBRs at 24 and 48 h after mice were injected with irradiated NPYR (Fig. 3). Injecting the mice with non-irradiated NPYR after 48 h of treatment was also genotoxic. Thus, the toxicity of metabolically activated NPYR via P450 might have also contributed to the MN formation (Fig. 3a). When the activity of P450 s was suppressed by ABT, MNs did not form in PBRs in mice injected with non-irradiated NPYR. However, the number of PBRs significantly increased after treatment with irradiated NPYR and ABT (Fig. 3b). After 24 h, the mutagenicity of the irradiated NPYR decreased to 32% at 37 ºC (Fig. 1a). The frequencies of MN was significantly increased in the mice untreated with ABT 48 h after injection of NPYR (Fig. 3a) but was not increased in ABT-treated mice (Fig, 3b) compared with those after 24 h of injection. Most of the genotoxicity of irradiated NPYR with ABT treatment was considered to occur during the first 24 h. Thus, MN formation in PBRs in mice treated with ABT was not significantly increased at 48 h compared to that at 24 h (Fig. 3b). Without ABT treatment, the genotoxicity of photoactivated-NPYR at 48 h was close to the sum of the genotoxicity of photoactivated-NPYR at 24 h and that of non-irradiated NPYR at 48 h, indicating that the toxicity of metabolically activated NPYR via P450 (Fig. 3a). The active derivative of NPYR which might be *N*-nitroso-1-phosphonooxypyrrolidine produced genotoxicity in bone marrow, resulting in MN formation in PBRs.

The action spectrum of induced MN formation followed the absorption curve of NPYR (Fig. 4). The production ratio of the active derivative of NPYR, *N*-nitroso-1-phosphonooxypyrrolidine, followed the absorption curve of NPYR, whereas the in vivo genotoxicity of NPYR irradiated with monochromatic light followed the absorption curve of NPYR. Wavelength-dependent genotoxicity suggested that the sensitization of NPYR by UVA triggers the formation of an active derivative of NPYR (Fig. 4).

Systemic genotoxicity occurs if externally irradiated NPYR penetrates the skin, or if penetrated NPYR was irradiated just under the skin to create irradiated NPYR, and has entered the systemic circulation. Risk analyses of public-health-related volatile *N*‐nitrosamines, including NPYR, via environmental photoactivation should be considered.

## Data Availability

No datasets were generated or analysed during the current study.

## Abbreviations

ABT: 1-Aminobenzotriazole
DMEM: Dulbecco’s Modified Eagle’s medium
ENU: N-Ethyl-N-nitrosourea
FBS: Fetal bovine serum
IP: Intraperitoneal
MNU: *N*-Methyl-*N*-nitrosourea
MN: Micronuclei
NDMA: *N*-Nitrosodimethylamine
NPYR: *N*-Nitrosopyrrolidine
OECD: Organization for Economic Co-operation and Development
PBRs: Peripheral blood reticulocytes
PBS: Phosphate buffered saline
SD: Standard deviation
